# Exposure-survival analyses of pazopanib in renal cell carcinoma and soft tissue sarcoma patients: opportunities for dose optimization

**DOI:** 10.1007/s00280-017-3463-x

**Published:** 2017-10-19

**Authors:** R. B. Verheijen, L. E. Swart, J. H. Beijnen, J. H. M. Schellens, A. D. R. Huitema, N. Steeghs

**Affiliations:** 1 Department of Pharmacy and Pharmacology, The Netherlands Cancer Institute - Antoni van Leeuwenhoek, Louwesweg 6, 1066 EC Amsterdam, The Netherlands; 2 Department of Pharmaceutical Sciences, Utrecht University, Utrecht, The Netherlands; 3 Department of Medical Oncology and Clinical Pharmacology, The Netherlands Cancer Institute - Antoni van Leeuwenhoek, Amsterdam, The Netherlands; 4 Department of Clinical Pharmacy, University Medical Center Utrecht, Utrecht University, Utrecht, The Netherlands

**Keywords:** Pazopanib, Renal cell carcinoma, Soft tissue sarcoma, Pharmacokinetics, Dose optimization, Personalized medicine

## Abstract

**Background:**

Pazopanib is an angiogenesis inhibitor approved for the treatment of renal cell carcinoma and soft tissue sarcoma. Post hoc analysis of a clinical trial demonstrated a relationship between pazopanib trough concentrations (C_min_) and treatment efficacy. The aim of this study was to explore the pharmacokinetics and exposure-survival relationships of pazopanib in a real-world patient cohort.

**Patients and methods:**

Renal cell cancer and soft tissue sarcoma patients who had at least one pazopanib plasma concentration available were included. Using calculated C_min_ values and a threshold of > 20 mg/L, univariate and multivariate exposure-survival analyses were performed.

**Results:**

Sixty-one patients were included, of which 16.4% were underexposed (mean C_min_ < 20 mg/L) using the 800 mg fixed-dosed schedule. In univariate analysis C_min_ > 20 mg/L was related to longer progression free survival in renal cell cancer patients (34.1 vs. 12.5 weeks, *n* = 35, *p* = 0.027) and the overall population (25.0 vs. 8.8 weeks, *n* = 61, *p* = 0.012), but not in the sarcoma subgroup (18.7 vs. 8.8 weeks, *n* = 26, *p* = 0.142). In multivariate analysis C_min_ > 20 mg/L was associated with hazard ratios of 0.25 (*p* = 0.021) in renal cancer, 0.12 (*p* = 0.011) in sarcoma and 0.38 (*p* = 0.017) in a pooled analysis.

**Conclusion:**

This study confirms that pazopanib C_min_ > 20 mg/L relates to better progression free survival in renal cancer and points towards a similar trend in sarcoma patients. C_min_ monitoring of pazopanib can help identify patients with low C_min_ for whom individualized treatment at a higher dose may be appropriate.

## Introduction

Pazopanib is an angiogenesis inhibitor, targeting the vascular endothelial growth factor receptor (VEGFR)-1,2,3, platelet derived growth factor receptor (PDGFR) α/β, fibroblast growth factor receptor and the stem cell receptor/ c-Kit [1, 2].

Pazopanib increased progression free survival in renal cell carcinoma and in soft tissue sarcoma compared to placebo, resulting in market approval for both tumor types by the Food and Drug Administration and European Medicine Agency [3, 4]. A retrospective analysis from clinical trial data showed an increased median progression free survival in patients with pazopanib plasma trough concentrations (C_min_) ≥ 20.5 mg/L compared to patients with lower concentrations (52.0 vs. 19.6 weeks, *n* = 177, *p* = 0.004) [5].

Pazopanib has a complex pharmacokinetic profile, described by low, non-linear and time-dependent bioavailability and large inter-individual variability [6–9]. This results in a subset of patients receiving less than optimal exposure [5]. It has been estimated in clinical trials that on the approved 800 mg dose, approximately 20% of patients may not reach the > 20 mg/L threshold [5].

In routine clinical care pharmacokinetic variability may be even greater, as patients are likely to have more comorbidities and concomitant medication, may be older, have impaired renal or hepatic function and have suboptimal therapy adherence [10]. In particular the elderly are known to be underrepresented in clinical trials [11]. Moreover, it has been reported that only 39.0% of renal cancer patients treated with targeted therapies in routine clinical practice would be eligible for enrolment in the pivotal phase III trials of their respective therapy [12].

The above underscores the need for exploration of the proportion of patients that risk suboptimal efficacy due to low exposure in real-word patient cohorts. Especially, since it has been shown that increasing the pazopanib dose based on a low C_min_ is a feasible and safe option that could lead to improved treatment outcomes [13].

We now report an observational unselected cohort study in renal cell carcinoma and soft tissue sarcoma patients to identify the number of patients at risk of suboptimal treatment due to low exposure. Additionally, we perform exposure–response and exposure-toxicity analyses and explore if patient characteristics could predict the occurrence of low pazopanib C_min_.

## Materials and methods

### Patient inclusion and data collection

An observational study was performed in the outpatient clinic of the Netherlands Cancer Institute, Amsterdam, The Netherlands. Plasma sampling for concentration monitoring was performed as part of routine care in all patients treated with pazopanib at this hospital (however, no dose increments above 800 mg based on low C_min_ were performed during the study period). In the current study, data from routine clinical care including pazopanib plasma concentrations were used retrospectively, which has been authorized in the institute.

Renal cell carcinoma and soft tissue sarcoma patients who received pazopanib treatment as part of standard of care and who had at least one pazopanib plasma concentration measured were included.

Visits were planned according to standard of care in accordance with respective treatment guidelines. Clinical characteristics including demographic data, medical history, pazopanib dose, treatment duration, reason for discontinuation and progression free survival were collected retrospectively from medical records.

### Pharmacokinetics

Blood samples were drawn at routinely scheduled visits to the outpatient clinic. Date and time of last intake of pazopanib dose and the time of blood sampling were recorded. Plasma pazopanib levels were determined using a validated liquid chromatography tandem mass spectrometry assay [14]. C_min_ values were calculated based on the measured concentration and interval between last ingested dose and sample time using the algorithm developed previously for imatinib [15]. Samples drawn before T_max_ (2 h) [8] or more than 24 h after the last dose were excluded from the analysis.

Relationships between C_min_ and available patient characteristics were explored, including tumor type, age, weight, gender, (lowest) pazopanib dose and World Health Origination (WHO) performance status. Binary variables were tested using two-sided* t* tests, categorical variables using analysis of variance, numerical variables using linear regression. *p* values < 0.05 were considered significant. All statistical analyses were performed in R 3.3.1 [16].

### Exposure-survival analysis

For the purpose of exposure-survival analyses the mean of all available C_min_ levels per patient was used as parameter for exposure during the entire treatment period, as described previously [13]. Progression free survival of patients with a mean C_min_ above or below the pharmacokinetic threshold of > 20 mg/L was analyzed in univariate (Kaplan–Meier analysis plus log-rank test) and multivariate analyses using Cox regression. In multivariate analysis performances status, (lowest) pazopanib dose, number of prior lines of therapy, age and sex were included as covariates. For the exposure-survival analyses in sarcoma, the tumor subtype (leiomyosarcoma, synovial sarcoma or other) was included as an additional covariate. Results are reported as hazard ratios plus 95% confidence intervals (95% CI). A pooled analysis of all patients was also performed. Here, tumor type (renal cancer versus sarcoma) was also included in the multivariate Cox regression.

### Exposure-toxicity analysis

Pharmacokinetic exposure was compared between patients who discontinued pazopanib therapy due to toxicity and those who did not. Both the average C_min_ per patient and the last C_min_ closest to the discontinuation event (due to toxicity or progressive disease) were analyzed.

## Results

### Evaluable patients

From April 2013 to November 2016, 61 patients were included in the analysis, of whom 35 had renal cell carcinoma and 26 soft tissue sarcoma. A full overview of patient characteristics, including WHO performance status, pazopanib dose, previous lines of therapy, age, weight, sex and number of samples is given in Table 1.

**Table 1:** Characteristics of included patients

	Renal cell carcinoma	Soft tissue sarcoma	Overall
Patients (*n*)	35	26	61
Gender (*n* (%))			
Male	22 (62.9)	14 (53.8)	36 (59.0)
Female	13 (37.1)	12 (46.2)	25 (41.0)
Age (mean (range))	62 (45–77)	61 (32–91)	61 (32–91)
Weight (mean (CV%))	84 (23.1)	77 (17.5)	81 (21.6)
Performance status (*n* (%))			
0	13 (37.1)	11(42.3)	24 (39.3)
1	16 (45.7)	14 (53.8)	30 (49.2)
2	6 (17.1)	1 (3.8)	7 (11.5)
Pazopanib dose (*n* (%))*			
200 mg	3 (8.6)	1 (3.8)	4 (6.6)
400 mg	5 (14.3)	2 (7.7)	7 (11.5)
600 mg	6 (17.1)	2 (7.7)	8 (13.1)
800 mg	21 (60.0)	21 (80.8)	42 (68.9)
Previous lines of systemic therapy (median (range))	1 (1–4)	1 (0–2)	1 (0–4)
Number of samples (*n*)	151	76	227
Samples per patients (mean (range))	4 (1–17)	3 (1–9)	4 (1–17)
Mean (CV%) C_min_ per patient (mg/L)	26.9 (36.4)	31.9 (36.3)	29.0 (37.1)
Patients with mean C_min_ < 20 mg/L (*n* (%))	6 (17.1)	4 (15.4)	10 (16.4)

*C*_*min*_ Pazopanib trough level/minimum concentration, *CV%* coefficient of variation

*Lowest dose per patient

The subtypes of the sarcoma patients included leiomyosarcoma (*n* = 12), synovial sarcoma (*n* = 6), pleomorphic sarcoma (*n* = 2), epithelioid sarcoma, malignant peripheral nerve sheath tumor, angiosarcoma, solitary fibrous tumor, myxofibrosarcoma and undifferentiated spindle cell sarcoma (all *n* = 1).

### Pharmacokinetics

In total, 227 plasma samples were included. Overall, a mean (range) of 4 (1–17) samples were available per patient, 3 (1–9) for the sarcoma and 4 (1–17) for the renal cancer patients. In aggregate, mean (coefficient of variation (CV%)) pazopanib C_min_ was 28.1 (39.7) mg/L, ranging from 6.90 to 77.8 mg/L. Median [range] sampling time was 6 [1–44] months since start of therapy. With 5% of samples taken < 4 weeks after start. Median interpatient variability (quantified as CV% of the multiple C_min_ values per patient on the same dose) was 24.8%.

**Fig. 1:**
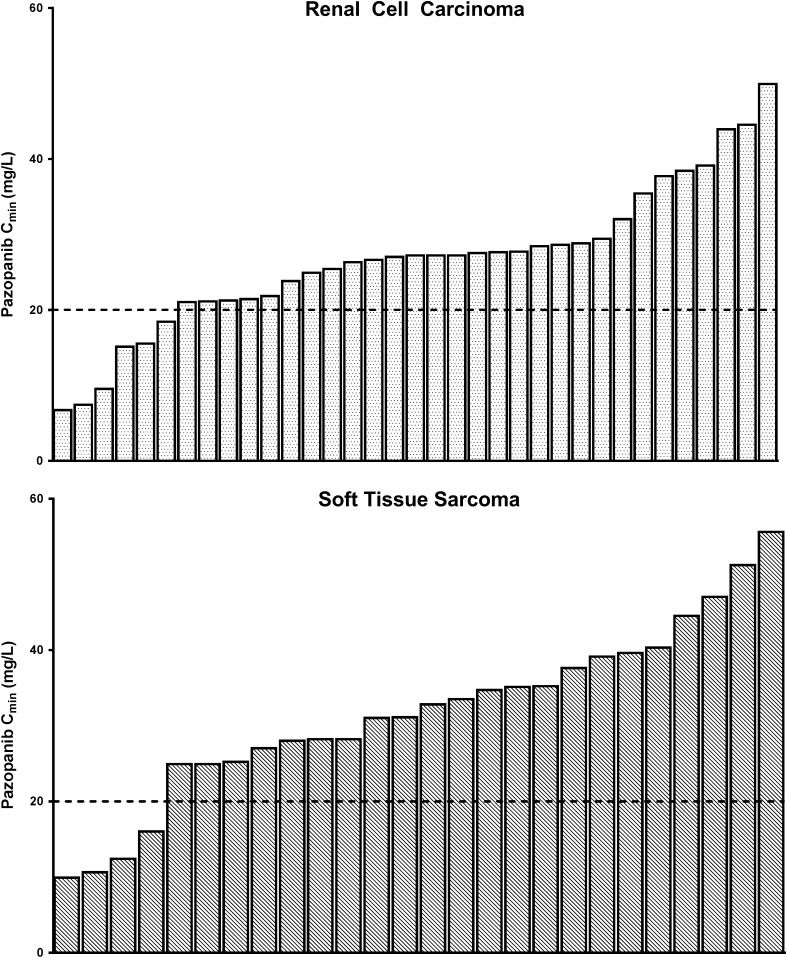
Distribution of the mean calculated pazopanib C_min_ per patient for renal cell carcinoma *n* = 35 (upper panel) and soft tissue sarcoma patients *n* = 26 (lower panel). The dotted line indicates the threshold of 20 mg/L. In renal cell carcinoma 6 (17.1%) of patients and in soft tissue sarcoma 4 (15.4%) of patients seem underexposed using the 800 mg fixed-dosed schedule

An overview of the distribution of average C_min_ per patients per tumor type is provided in Fig. 1 and Table 1. In renal cancer patients mean (CV%) C_min_ per patient was 26.9 (36.4) mg/L compared to 31.9 (36.3) mg/L in the sarcoma patients. The overall average C_min_ per patient was 29.0 (37.1) mg/L.

Although C_min_ was higher in sarcoma compared to renal cancer patients (Table 1), this difference was not statistically significant (*p* = 0.081). In renal cell carcinoma 6 (17.1%) and in soft tissue sarcoma 4 (15.4%) patients were underexposed (mean C_min_ < 20 mg/L) using the 800 mg fixed-dose schedule.

Of all explored clinical parameters, none were found to be significantly predictive of low pazopanib C_min_ except gender in renal cell cancer patients and age in sarcoma patients. Female sex was associated with a higher C_min_ (mean (CV%) of 33.1 (32.5) mg/L versus 23.2 (30.1) *p* = 0.005). Of the six renal cancer patients with low C_min_ only one was female.

In sarcoma patients, linear regression indicated that patients with higher age had lower C_min_ and was associated with a slope of − 0.454 and Pearson’s r of − 0.414 (*p* = 0.035).

### Exposure-survival analysis

**Fig. 2:**
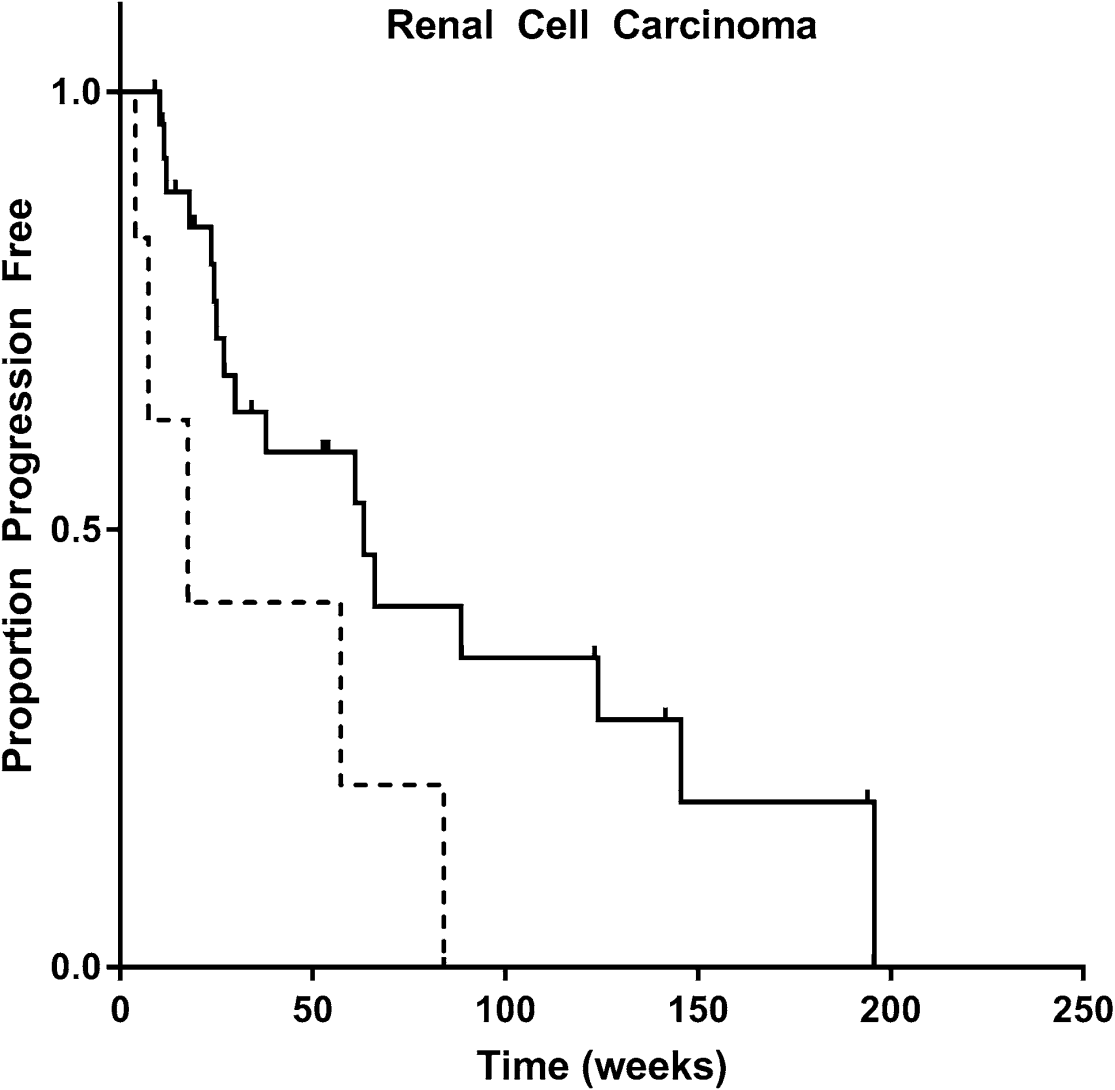
Kaplan–Meier plot of progression free survival (weeks) for renal cell carcinoma patients with an average C_min_ above (*n* = 29, solid line) or below (*n* = 6, dashed line) the exposure target of > 20 mg/L. Median progression free survival was 34.1 weeks for patients with high and 12.5 weeks for patients with low exposure, *p* = 0.027 (log-rank test)

In renal cell carcinoma, C_min_ > 20 mg/L was significantly related to improved progression free survival in univariate analysis (*p* = 0.027, see Fig. 2 and Table 2). Median progression free survival was 34.1 weeks for patients with high and 12.5 weeks for patients with low exposure.

**Table 2:** Overview of exposure-survival analysis outcomes

	Renal cell carcinoma	Soft tissue sarcoma	Overall
Patients (*n*)	35	26	61
Median PFS (weeks)	29.9	18.3	24.4
Median PFS C_min_ > 20 mg/L (weeks)	34.1	18.7	25.0
Median PFS C_min_ < 20 mg/L (weeks)	12.5	8.8	8.8
Univariate *p* value (log-rank test)	0.027	0.142	0.012
Hazard ratio (95% CI)*	0.25 (0.076–0.81)	0.12 (0.024–0.61)	0.38 (0.17–0.92)
Multivariate *p* value (Cox regression)	0.021	0.011	0.017

*C*_*min*_ pazopanib trough level/minimum plasma concentration

*PFS* progression free survival

*95% CI* 95% confidence interval

*Hazard ratios are based on the multivariate Cox regression analysis

In multivariate analysis, C_min_ above or below 20 mg/L resulted in a hazard ratio of 0.25 (95% CI 0.076–0.81, *p* = 0.021). Female gender was also significantly related to increased progression free survival (*p* = 0.008).

**Fig. 3:**
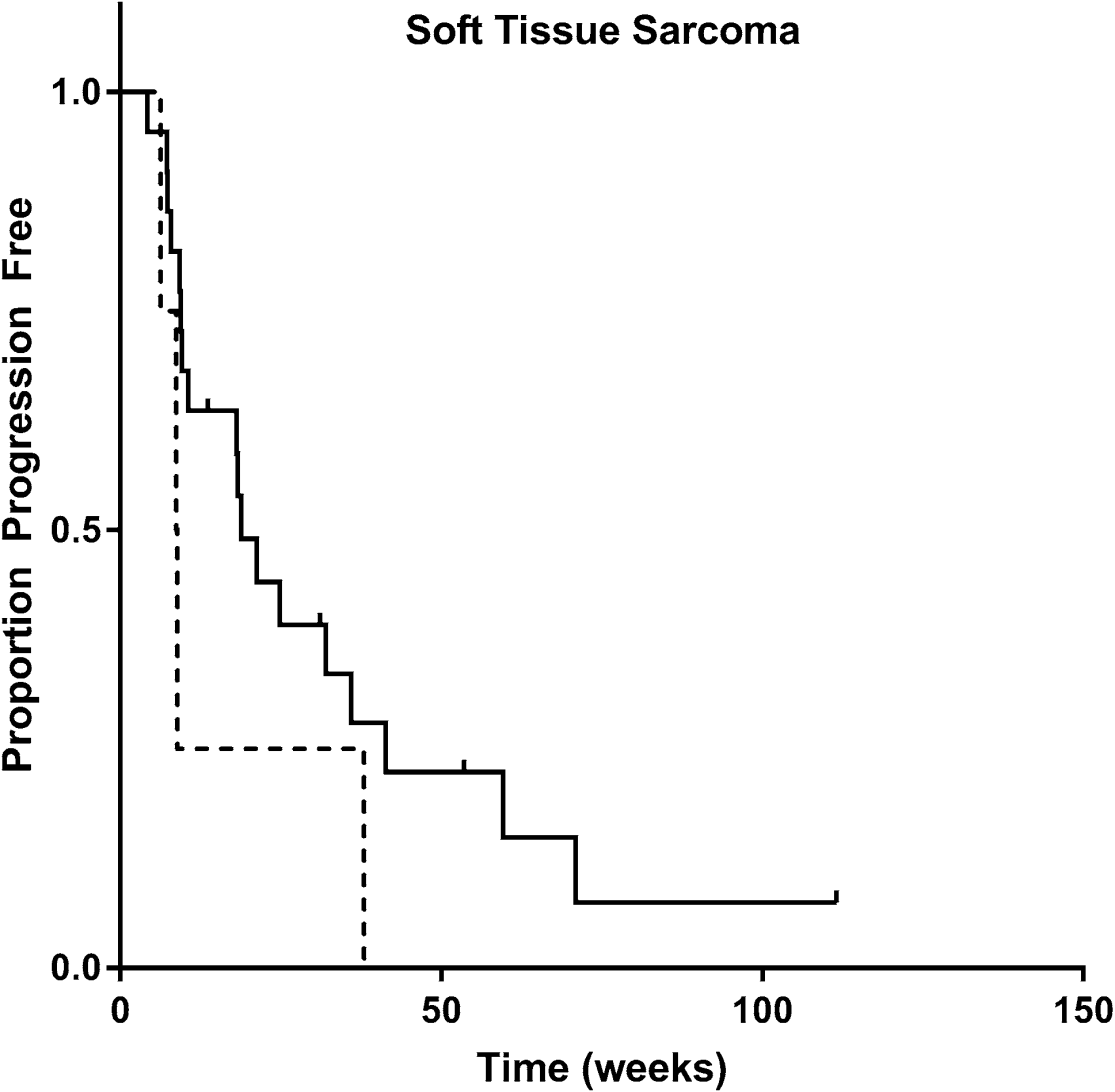
Kaplan–Meier plot of progression free survival (weeks) for soft tissue sarcoma patients with an average C_min_ above (*n* = 22, solid line) or below (*n* = 4, *dashed line*) the exposure target of > 20 mg/L. Median progression free survival was 18.7 weeks for patients with high and 8.80 weeks for patients with low exposure, *p* = 0.142 (log-rank test)

In soft tissue sarcoma, median progression free survival was 18.7 weeks for patients with high and 8.8 weeks for patients with low C_min_ (*p* = 0.142, log-rank test, see Fig. 3; Table 2). In Cox regression, C_min_ > 20 mg/L was significantly related to progression free survival and associated with an hazard ratio of 0.12 (95% CI 0.024–0.61, *p* = 0.011). In the sarcoma subgroup, worse performance status (*p* = 0.035) and lower age (*p* = 0.017) were also associated with shorter progression free survival.

**Fig. 4:**
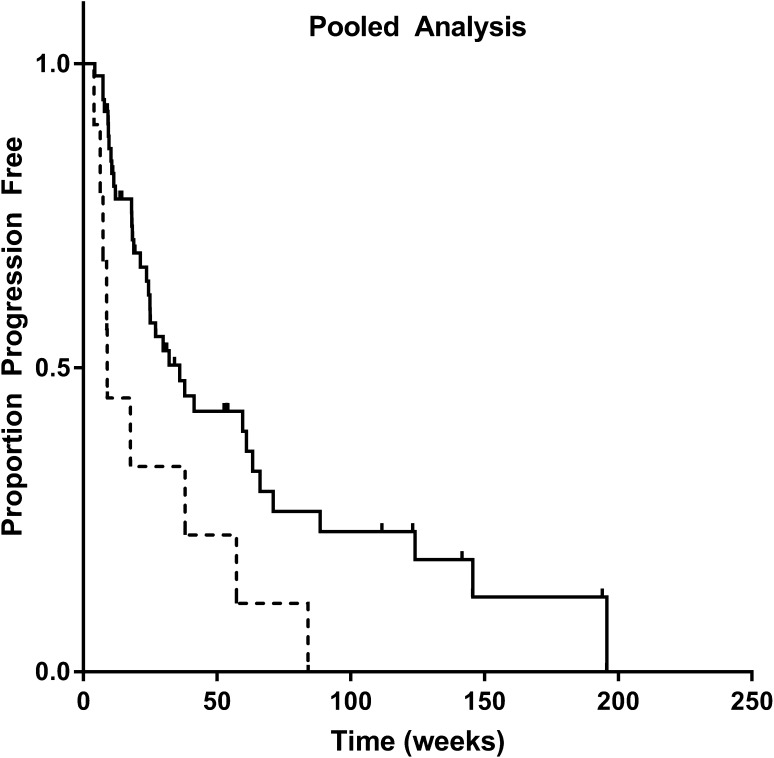
Kaplan–Meier plot of progression free survival (weeks) in a pooled analysis of both renal cell carcinoma and soft tissue sarcoma patients with an average C_min_ above (*n* = 51, solid line) or below (n = 10, dashed line) the exposure target of > 20 mg/L. Median progression free survival was 25.0 weeks for patients with high and 8.80 weeks for patients with low exposure, *p* = 0.012 (log-rank test)

In the pooled analysis, C_min_ > 20 mg/L was related to improved survival in univariate analysis (25.0 vs. 8.8 weeks, *p* = 0.012, see Fig. 4 and Table 2). Here, multivariate analysis resulted in a hazard ratio of 0.38 (95% CI 0.17–0.92, *p* = 0.017) for C_min_ > 20 mg/L. Worse WHO performance status and sarcoma as tumor type were also both associated with worse treatment outcome in the Cox model, *p* = 0.004 and *p* < 0.001, respectively.

An overview of the main univariate and multivariate exposure-survival outcomes is provided in Table 2.

### Exposure-toxicity analysis

Of the 61 included patients, 44 discontinued treatment due to progressive disease and 5 due to toxicity. Reasons for discontinuation included, hepatotoxicity, hypertension, pancreatitis, dyspnea and multiple grade 2 toxicities (all *n* = 1). Mean C_min_ was 37.3 mg/L in those who discontinued due to toxicity, compared to 27.5 mg/L in those had experienced progressive disease. However, this difference was not statistically significant (*p* = 0.176). Mean (CV%) last C_min_ (the last available sample) was 37.7 (36.4) mg/L compared to 26.4 (46.9) mg/L (*p* = 0.177).

## Discussion

Pazopanib is administered at a fixed 800 mg dose, which is only adjusted in case of severe toxicity. Yet, based on the available data this may lead to suboptimal treatment outcomes in a subset of patients [5]. We now show in an unselected cohort that approximately 16.4% of patients is underexposed using the pazopanib fixed-dosing schedule applying the predefined C_min_ target of > 20 mg/L (Fig. 1, Table 1).

No clinical characteristics, except for gender and age were found to be significantly related to pazopanib C_min_. In general, the ability of clinical characteristics to predict which patients experienced low C_min_ was limited. This underscores the relevance of routine pazopanib C_min_ monitoring, as the subgroup at risk of lower efficacy cannot be identified employing clinical and demographic characteristics. Furthermore, the use of potential interacting medication is carefully monitored during routine care and, therefore, no effects of concomitantly used medication on PK exposure could be identified.

We demonstrate that in renal cancer patients C_min_ > 20 mg/L was significantly related to longer progression free survival (34.1 vs. 12.5 weeks, Table 2 and Fig. 2). Our data, therefore, confirm the findings of Suttle et al. in an independent patient cohort [5].

As no pharmacokinetic sampling was performed in the pivotal phase II trial in soft tissue sarcoma,[4] no pazopanib exposure threshold has been proposed yet in sarcoma. This is the first study to investigate a relationship between exposure and survival in sarcoma. However, possibly due to the limited size of the sarcoma subgroup in our cohort and the relatively lower effect size of pazopanib in sarcoma, our result did not reach statistical significance in univariate analysis (progression free survival of 18.7 vs. 8.8 weeks, *p* = 0.142, Fig. 3). Another possible explanation for the lack of significance in the univariate exposure-survival analysis could be the diversity of sarcoma subtypes. This heterogeneity may, therefore, explain differences in response rates and response duration between disease subtypes. However, in the multivariate analysis in sarcoma this difference in progression free survival for patients with C_min_ > 20 mg/L was statistically significant (*p* = 0.011).

In a pooled exposure-survival analysis (Fig. 4) higher pazopanib C_min_ was significantly related to improved treatment outcomes. Furthermore, the existence of a similar exposure–response relationship is theoretically supported by the fact that efficacy of pazopanib is mediated by inhibition of the same target proteins (mainly VEGFR) in both tumor types. However, this exploratory pooled analysis should be interpreted with caution given the variability in sensitivity of the different tumor types.

Previous exposure-toxicity relationships have been reported for pazopanib related adverse events such as hepatotoxicity and hypertension and dose-limiting toxicity in pediatric patients [5, 17, 18]. Although not statistically significant, in this cohort we did find a numerically higher exposure in patients discontinuing due to toxicity (*n* = 5), this result was not statistically significant (37.3 vs. 27.5 mg/L, *p* = 0.176).

Drawbacks of this study are its retrospective nature, relatively limited number of patients in each tumor type and the heterogeneity in the availability and timing of plasma samples. Furthermore, not actual but calculated C_min_ values (using an therapeutic drug monitoring algorithm as validated for imatinib) were used. However, this algorithm describes a general exponential decline in exposure with a specified plasma half-life and would, therefore, also be suitable for pazopanib.

Yet despite these limitations, it is the first pharmacokinetic study that reports exposure-survival and exposure-toxicity relationships for pazopanib in a real-world cohort of renal cell carcinoma and soft tissue sarcoma patients and identifies a subgroup of approximately 16.4% of patients which may benefit from individualized C_min_-guided pazopanib dosing.

Given the currently presented results and the previous work by Suttle et al [5], one could argue that a fixed dosing strategy for pazopanib is becoming increasingly inappropriate in the era of personalized medicine [19, 20]. Recommendations for individualized dosing of pazopanib and other tyrosine kinase inhibitors have been made previously [21, 22]. Moreover, specifically for pazopanib the safety and feasibility of individualized dosing has been established in a prospective clinical trial [13].

Collectively, the current study and data available in the literature [5, 13] point towards the need to validate the strategy of individualized pazopanib dosing in a prospective randomized clinical trial.

## Conclusion

In conclusion, at the currently approved fixed dose regimen a relevant subgroup of 16.4% of patients treated with pazopanib is underexposed in routine care and may be at risk of suboptimal treatment efficacy.

Our study further confirms that the previously established threshold of C_min_ > 20 mg/L is related to longer progression free survival in renal cell carcinoma patients (34.1 vs. 12.5 weeks, *n* = 35, *p* = 0.027). Moreover, exploratory analyses point towards a similar association of increased progression free survival with higher exposure in soft tissue sarcoma patients (18.7 vs. 8.8 weeks, *n* = 26, *p* = 0.142).

Plasma C_min_ monitoring of pazopanib can help identify patients with low C_min_ for whom treatment at a higher dose may be appropriate.
